# Protein Recognition in Drug-Induced DNA Alkylation: When the Moonlight Protein GAPDH Meets S23906-1/DNA Minor Groove Adducts

**DOI:** 10.3390/ijms161125971

**Published:** 2015-11-05

**Authors:** Gaëlle Savreux-Lenglet, Sabine Depauw, Marie-Hélène David-Cordonnier

**Affiliations:** UMR-S1172—Jean-Pierre Aubert Research Centre (JPARC), INSERM, University of Lille, Lille Hospital, Institut pour la Recherche sur le Cancer de Lille, Place de Verdun F-59045 Lille cedex, France; gaelle.lenglet@u-picardie.fr (G.S.-L.); sabine.depauw@inserm.fr (S.D.)

**Keywords:** DNA alkylation, DNA repair, Glyceraldehyde-3-phosphate dehydrogenase, S23906-1, protein/DNA binding

## Abstract

DNA alkylating drugs have been used in clinics for more than seventy years. The diversity of their mechanism of action (major/minor groove; mono-/bis-alkylation; intra-/inter-strand crosslinks; DNA stabilization/destabilization, *etc.*) has undoubtedly major consequences on the cellular response to treatment. The aim of this review is to highlight the variety of established protein recognition of DNA adducts to then particularly focus on glyceraldehyde-3-phosphate dehydrogenase (GAPDH) function in DNA adduct interaction with illustration using original experiments performed with S23906-1/DNA adduct. The introduction of this review is a state of the art of protein/DNA adducts recognition, depending on the major or minor groove orientation of the DNA bonding as well as on the molecular consequences in terms of double-stranded DNA maintenance. It reviews the implication of proteins from both DNA repair, transcription, replication and chromatin maintenance in selective DNA adduct recognition. The main section of the manuscript is focusing on the implication of the moonlighting protein GAPDH in DNA adduct recognition with the model of the peculiar DNA minor groove alkylating and destabilizing drug S23906-1. The mechanism of action of S23906-1 alkylating drug and the large variety of GAPDH cellular functions are presented prior to focus on GAPDH direct binding to S23906-1 adducts.

## 1. Introduction

The concept of chemotherapy to treat cancer was born with the experiments of the famous chemist Prof P. Ehrlich at the early 20th century with evaluation of aniline dyes (initially synthesized by Prof. W.H. Perkin as early as 1856) and ethyleneimine as a primitive alkylating compound [1]. The first synthesized alkylating agent was *bis*-(β-chloroethyl)-sulfide in the mid-nineteenth century as a precursor of other sulfur mustards. Those sulfur mustards are considered the first anti-cancer agents from the initial observation of the leukopenic effect (decrease of lymph nodes and bone marrow cell number) in soldiers that were exposed to mustard gas during World War I and further accidentally exposed troops after the explosion of military stocks of mustard gas in December 1943. Consequently, sulfur and nitrogen mustards were evaluated at Yale University for potential therapeutic effects. Chemotherapeutic use of mustards was first evaluated as early as 1942 on a patient (named from his initials “JD”) presenting a radiotherapy-resistant lymphosarcoma at relapse two years post-surgery and radiation therapy. Results showed interesting antitumor activity but the primary promising reduction of nodes was rapidly followed by relapse associated with chemoresistance (reviewed in [2]). Since this pioneer period, mustards are still used in cancer treatment (chlorambucil, melphalan) but anti-cancer chemotherapy was, thankfully, largely extended to better target cancer cells with a much wider pharmacopeia including a large variety of drugs from several families of alkylating agents and then by new strategies of targeted therapies. However, because of frequent chemoresistance (either at relapse after conventional and targeted chemotherapies or as primary chemoresistance observed in some cancer subtypes such as pancreatic cancer, kidney carcinoma, glioblastoma, *etc.*) or because of the absence a clear target yet identified in all subtypes of cancer, it remains important to develop original new anti-cancer drugs, such as new DNA alkylating compounds. It is also crucial to get further information about their precise mechanism of action and their cellular response potentially leading to chemoresistance and relapse. This is particularly true in terms of DNA repair of DNA alkylating drugs. Particularly, a better knowledge of proteins being implicated in adduct recognition and how they could modulate the cellular effects of the alkylating drug treatment are therefore of major interest in new cancer drug development. The aim of the present study is therefore first to give an overview on protein/DNA adduct recognition as an introduction to then focus on the glyceraldehyde-3-phosphate dehydrogenase (GAPDH) cellular functions and its particular role on protein recognition of DNA damage and its recently discovered implication in DNA repair. This latter part is illustrated with the particular example of the S23906-1 DNA adduct formed on either double- or single-stranded DNA or on telomeric DNAs and its recognition by GAPDH.

### 1.1. DNA Alkylation and Destabilization by DNA Alkylating Drugs

#### 1.1.1. DNA Major Groove Alkylating Agents

Within the alkylating drugs family, it is worth noting that most of the “old drugs” target the DNA though a covalent bonding linking the molecule to the major groove of the DNA helix (mustards, nitrosourea, platinated agents, mitomycines, *etc.*).

**Figure 1:**
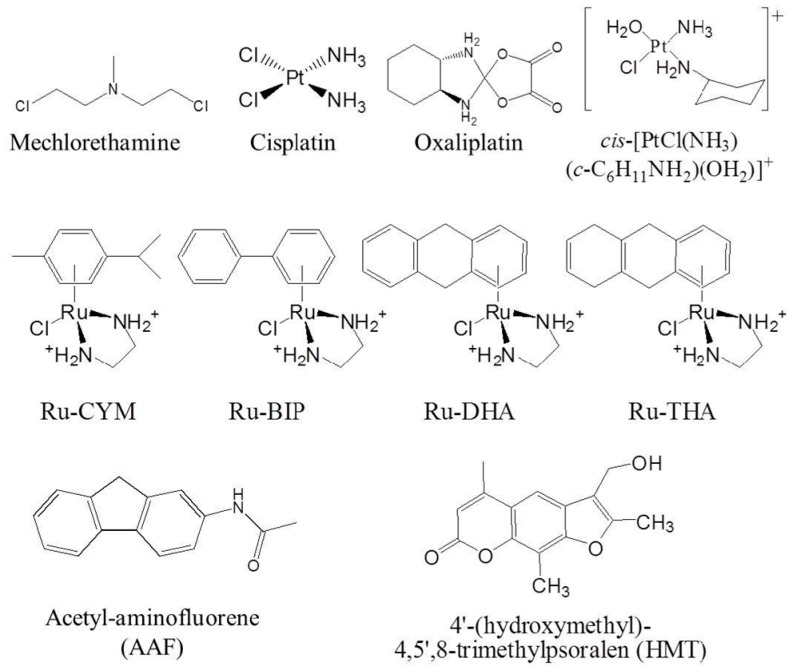
Structures of the DNA major groove alkylating agents presented in this review.

For instance, nitrogen mustards react mainly with the N7 position of guanine residues [3,4] through an intermediary aziridinium to form an N7-alkylated guanine orientated toward the major groove of the DNA helix. This mono-adduct could then be converted to a second intermediate aziridinium, which may either react with water or with a second guanine residue to finally form the interstrand cross-link at 5′-CNG sequence [3,4,5]. In term of global three-dimensional DNA orientation, structural studies shows that mechloretamine (Figure 1) crosslinks at 5′-CNG is associated with a stabilization of the DNA helix and induces only a weak curvature of the DNA axis by 14° is observed [6].

By contrast, some platinum derivatives are able to destabilize the DNA helix in correlation with a strong distortion of the DNA axis. This is the case for cisplatin (Figure 1) for which the induced intra-strand crosslinks at 5′-GG base pairs result in a 55–78° bending of the DNA axis toward the major groove and allows the local denaturation of the DNA helix through the destabilization of Watson–Crick base pairing [7,8]. The distortion by platinated-GG intra-strand crosslinks greatly depends on the sequence context. As is, for example, a decrease of the melting temperature of more than 10 °C with cisplatin adducts occurring at 5′-TGGT sequence from comparison to a 6 °C decrease using 5′-CGGT and 5′-AGGC bonding sites [9,10], and up to a seven base-pairs destabilization for 1,3-intrastrand crosslink in a 5′-TGTGT site [11]. In an interesting manner, oxaliplatin (Figure 1), the third-generation of platinum derivative commonly used in clinic, induces greater DNA bending, unwinding and helix destabilization than cisplatin. This correlates with weaker High Mobility Group (HMG) proteins recognition of oxaliplatin- over cisplatin-induced lesions [11], suggesting a modified efficiency of DNA repair processes because of the widening of the locally destabilized portion of the DNA helix. It is also worth noting that DNA destabilization propensity is not a propensity that is shared by all platinium derivatives. Indeed, the pyrazolato-bridged dinuclear platinum(II) complex [(*cis*-{Pt(NH_3_)_2_})_2_(mu-OH)(mu-pyrazolate)]^2+^ (Figure 1), a bifunctional platinated derivatives which cross-links two adjacent guanines, unwinds DNA by ~15° with no change of the DNA helix axis and no DNA destabilization [12].

Other transition-metal antitumor agents also alkylate guanines in the major groove at N7 position and unwind DNA as using Ru-CYM, Ru-BIP, Ru-DHA, Ru-THA and monodentate-Ru(II) [13,14,15]. This is the case of acetyl-aminofluorene (AAF) (Figure 1) that reduced by 12 to 18 °C the melting temperature of different double stranded DNAs [16,17].

DNA destabilization by major groove targeting drugs is not only associated with alkylation at guanine residues but could also occur on two adjacent thymine residues as for the psoralen derivative 4′-hydroxymethyl-4,5′,8-trimethylpsoralen (HMT) (Figure 1) [18] (for further details see reviews [19,20]).

#### 1.1.2. DNA Minor Groove Alkylating Agents

**Figure 2:**
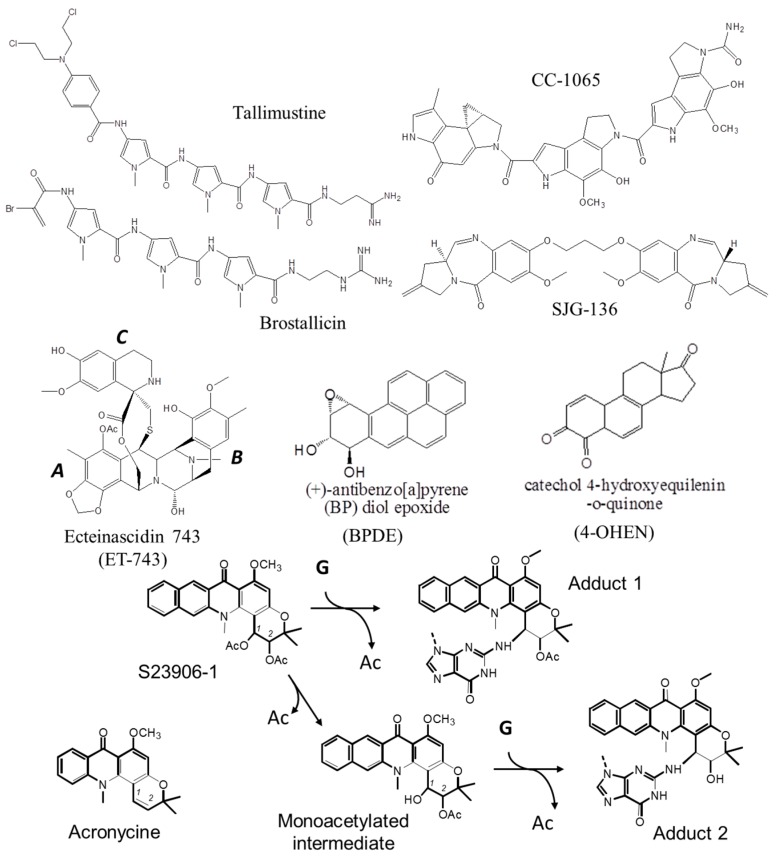
Structures of the DNA minor groove alkylating agents described in the text and S23906-1/DNA alkylation process. The covalent reaction with a guanine (“G”) leads to a concomitant release of one acetate group (“Ac”).

In contrast to the vast number of major groove alkylating agents depicted in literature, only a few compounds target the DNA helix through covalent bonding orientated in the minor groove. This is the case for the clinically used drug Ecteinascidin 743 (ET-743, *Trabectedin*, *Yondelis*^TM^ (PharmaMar, Madrid, Spain and Janssen Biotech Inc., Horsham, PA, USA), or for the distamycin derivatives tallimustine (PNU152241) and brostallicin (PNU-166196, CTI BioPharma Corp., Seattle, WA, USA) [21,22], as well as for the cyclopropylindol CC-1065, the pyrrolobenzodiazepin dimer SJG-136 (NSC694501, Spirogen, London, UK) [23,24] and the benzoacronycine derivative S23906-1 (*cis*-1,2-diacetoxy-1,2-dihydro-benzo-[*b*]-acronycine, licensed by Servier) that entered either preclinical or phase I/II clinical trials (Figure 2). Both distamycin derivatives and CC-1065 target guanines or adenines through covalent bonding at the N3 position and subsequently bend the DNA axis in a manner that is associated with local stabilization of the double-stranded DNA helix [25]. Both ET-743, SJG-136 and S23906-1 covalently bond DNA at the exocyclic amino-group of guanines localized in the minor groove [23,24,26,27,28,29]. However, those NH_2_ adducts differ on the global 3D structure of the DNA helix: ET-743 and SJG-136 stabilize the DNA helix [23,30], whereas S23906 destabilizes the hydrogen bonds between the two DNA strands [30]. S23906-1 forms two types of adducts with guanines depending on deacetylation occurring during the guanine alkylation process (adduct 1) or reacting with water first (spontaneous deacetylation process to form a monoacetylated intermediate) followed by a transesterification process that then allows alkylation at guanine residues to form adduct 2 (Figure 2) [27,28,29].

DNA destabilization following minor groove bonding is also achieved by carcinogens (such as BPDE ((+/−)-anti-benzo[*a*]pyrene-7,8-dihydrodiol-9,10-epoxide) [31,32]) or hormonal derivatives (for instance the genotoxic 4-OHEN (4-hydroxyequilenin-*O*-quinone) alkylating compound (Figure 2) [33,34]).

### 1.2. Protein Recognition of DNA Adducts

Proteins that specifically recognize DNA adducts could affect their mechanism of action, either positively or negatively. Those proteins belong to different families: DNA repair proteins, DNA polymerases, transcription factors, structural chromatin-interacting proteins, *etc.* The selectivity of the protein/DNA recognition depends either on the nature of the DNA adduct, the orientation toward the DNA groove (major or minor) or the consequences of the adduct formation of the DNA structure (bending, intercalation, local opening). A few examples are presented below. The detection of such protein/DNA adduct recognition was performed using various experimental approaches, mostly electrophoretic mobility shift assays (EMSA) as well as chromatography purification and/or separative gel purification coupled with mass spectrometry.

#### 1.2.1. Protein Recognition of Major Groove DNA Adducts

The protein recognition was well studied using cisplatin and other platinated adducts and evidenced strong binding of chromatin-interacting proteins, transcription factors and DNA repair proteins.

First, platinated DNA is recognized by HMG protein as a consequence of the strong DNA-bending generated by the platinated agent that perfectly fit with the L-shape structure of HMG DNA binding domain (HMG-box). As a pre-bent structure, cisplatin/DNA adducts reduces the energy that is required for HMG to bend the DNA [35]. However, the intrinsic bending that results from DNA alkylation should be in the same order of magnitude as that required by the protein for its binding to DNA. Indeed, oxaliplatin, which induces a stronger DNA bending and subsequently a stronger DNA destabilization, was poorly recognized by HMG protein [36]. It is assumed that HMG binding protects the platinated adducts from DNA repair machinery [37] and therefore reinforces their cytotoxicity [38]. The much weaker HMG recognition of oxaliplatin adducts allows a weaker DNA damage protection from DNA repair processes. This results in a fewer number of oxaliplatin lesions quantified in cells treated by oxaliplatin from comparison with lesions induced by cisplatin. As another target of the N7 position of guanine in the major groove, HMG binding of DNA adduct is similarly observed for the AAF-DNA adduct [39].

Second, cisplatin/DNA adducts are good substrates for the HMG-box containing transcription factors SRY and LEF-1, affecting their transcriptional activities [40,41].

Third, regarding DNA repair machineries, nucleotide excision repair (NER) is implicated in major groove platinated adducts recognition and repair. Particularly, the replication protein A (RPA), which is a single-stranded DNA-binding protein implicated in the recognition of single-stranded DNA bearing bulky adducts [42,43], recognizes cisplatin adducts [44,45]. This recognition leads to the recent development of RPA inhibitors such as MCI13E or TDRL-551 that showed promising synergy with cisplatin in ovarian or lung cancer models [46,47]. RPA also recognizes ruthenium adducts but to a lesser extent than platinated adducts. Cisplatin adducts are also recognized by *Xeroderma Pigmentosum* complementation group A (XPA) as recently evidenced using its yeast homolog Rad14 [17]. Rad14 efficiently binds to DNA containing Y-junctions, bubbles and bulges containing DNA [48] but poorly binds to native double-stranded DNA. It takes advantage of the cisplatin-induced DNA bending toward the major groove to bind as a dimer by inserting a β-hairpin chain from its DNA binding domain six base pairs away from the cisplatin adduct in order to cover a global 13-mer recognition sequence on the DNA [17]. Similar recognition occurs with the AAF-adduct [16,17]. By contrast, local platinated derivatives-induced DNA destabilization due to poor base stacking, base flipping and kinking interferes with their recognition of by repair proteins [49,50,51] such as the MSH2/MSH6 heterodimer (MutSα) from the classical mismatch repair machinery. MutSα binds mismatched cisplatin crosslinks but not transplatin adducts that does not destabilize the DNA [52,53,54,55].

Finally, major groove DNA adducts could also inhibit DNA polymerase activity and subsequence DNA synthesis as evidenced in yeast using AAF [56].

The NER machinery is also affected by major groove ruthenium adducts but to a lesser extent than by the cisplatin adducts. Ru-CYM adducts are more efficiently excised than Ru-THA complex adducts, consistently with a greater DNA destabilizing activity of Ru-CYM and a stronger DNA binding of RPA to this adduct [57]. It is interesting to note here the correlation between the efficient excision and a strong DNA binding of RPA [57].

#### 1.2.2. Protein Recognition of Major Groove DNA Adducts

Two main types of DNA adducts were well studied for protein/DNA recognition: the adduct lesions induced by genotoxic benzo[*a*]pyrene (BaP) and those induced by the clinically used drug ET-743/DNA to form a bulky lesion. Both alkylating occurs on the exocyclic N2 position of guanines located in the minor groove of the DNA.

In prokaryotes, BaP lesions were recognized by the NER sensor protein UvrB by taking advantage of the lesion-induced local thermodynamic destabilization and the induced flipping of the base [58]. By contrast, these lesions were identified in eukaryotes by the NER “sensor” protein *Xeroderma Pigmentosum* complementation group C (XPC). As for XPA, XPC requires a pre-bent DNA that is facilitated by local conformational flexibility and destabilization of the base pairing [59,60,61]. Sequence-dependent repair was also observed using BaP and BPDE for which a DNAadduct formed at 5′-CG*GC site (G*, alkylated guanine) is more rapidly excised than at 5′-CGG*C site in cell-free human HeLa extracts [62]. The single-stranded DNA-binding protein RPA and HMGB proteins also recognized the DNA destabilizing adduct BPDE [39,42].

ET-743 was approved by FDA in February 2015 for clinical use to treat advanced soft tissue sarcoma including liposarcoma and leiomyosarcoma. This tetrahydroisoquinoline alkaloid, isolated from the tunicate *Ecteinascidia turbinata*, is a DNA minor groove binder [26] that bends DNA toward the major groove [63]. It is formed of three subunits A/B and C (Figure 2) with covalent bonding to the NH_2_ group of guanines through a reactive iminium intermediate on A subunit, whereas A and B are together directly involved in the sequence-specific DNA binding and the perpendicular C subunit protrudes out of the double helix [26,30,64]. This bulky C-domain favors the recognition of ET-743/DNA adducts by DNA repair proteins (such as *Xeroderma Pigmentosum* complementation group G (XPG)) or transcription factors (see below). Indeed, ET-743 adducts block XPG protein on the DNA to form “cytotoxic complexes” as those generated using topoisomerase poisoning drugs [65,66]. More precisely, ET-743/DNA adducts trap the XPG endonuclease protein, leading to an increase of the generated single strand breaks responsible for the anti-tumor activity of ET-743 [67]. Such protein/adduct complexes prevent transcription of various genes [68,69,70,71] but also induce the rapid degradation of the active RNA polymerase II complex in transcription-coupled-nucleotide excision repair (TC-NER) proficient, but not in TC-NER deficient cells [72]. As a consequence and contrasting with other DNA adducts such as cisplatin, NER-deficient cell lines are resistant to ET-743. Independently of TC-NER, protein binding to ET-743/DNA adducts also induces DNA double-strand breaks through “replication fork collapse” as classically obtained using topoisomerase/poisoning drug/DNA complexes. Based on this peculiar effect, ET-743 was evaluated in patients with advanced non-small-cell lung cancer (NSCLC) after platinum treatment where XPG is over-expressed [73] or with patients with advanced ovarian cancer where XPG is mutated [74].

As another difference with cisplatin adducts, ET-743 does not trap transcription factors to their cognate binding site on promoter but, conversely, impairs their DNA binding abilities as evidenced using NF-Y (Nuclear Factor Y) [68,75], SRF/TCF (Serum-Response Factor/Ternary Complex Factor) [68] or HMGA (High Mobility Group protein A) [76]. The inhibitory effects of some of those transcription factors were suggested to take part of the mechanism of action of ET-743: (i) the inhibition of NF-Y impairs, if not all NF-Y-controlled genes, at least the expression of the drug efflux pump MDR1 (Multi-Drug Resistance protein 1), thus increasing its efficiency on multidrug-resistant tumors [69], or HSP70 (Heat Shock Protein 70) [70]; and (ii) inhibition of HMGA binding on ATM (*Ataxia Telangiectasia* Mutated protein) promoter reduces its expression thus leading to a better sensitivity of the cells to ionizing radiations [76].

Finally, the moonlight protein GAPDH was unexpectedly found as another DNA adducts interacting protein. This is exemplified in the following section with the model of S23906-1/DNA adduct recognition.

## 2. Glyceraldehyde-3-Phosphate Dehydrogenase (GAPDH) Binding to Damaged DNA: The Example of S23906-1/DNA Adduct Recognition

### 2.1. S23906-1

S23906-1 is a semi-synthetic derivative of the natural alkaloid acronycine by addition of a benzene ring and two acetate groups (Figure 2). Acronycine (3,12-dihydro-6-methoxy-3,3,12-trimethyl-7*H*-pyrano-[2,3-c]-acridin-7-one) was discovered in 1948. This alkaloid was first isolated from the bark of the Australian *Rutaceae* shrub *Acronychia baueri Schott* (*Sarcomelicope simplicifolia* (*Endl.*) Hartley ssp. *simplicifolia*) [77]. Its molecular structure (Figure 2) was then identified by MacDonald and Robertson as a pyran ring angularly fused to an acridone [78,79]. Acronycine showed interesting *in vivo* antitumor activities against a broad spectrum of solid tumors, in particular on chemo-resistant tumors such as the 180 sarcoma, S-115 carcinoma, S-91 melanoma and X-5563 myeloma cell lines [80]. Because of its poor solubility in water and other biocompatible solvants, acronycine was administrated *per-os* to patients suffering from refractory multiple myeloma. However, limiting gastrointestinal and neurotic toxicities stopped its development [81].

At the molecular level, the mechanism of action of acronycine was not fully determined: some authors suggested possible reactivity of acronycine with DNA, since acronycine stabilized the DNA double helix upon thermal denaturation [82]. Further isolation of acronycine derivatives from other shrubs of *Sarcomelicope* (*Rutaceae*) identified the 1-2-epoxy-acronycine derivative as a possible *in vivo* bioactivated intermediate [83]. Unfortunately, the chemical instability of the epoxide made it impossible to use as an antitumor agent. Many other derivatives of natural origin have been subsequently isolated, and many derivatives were synthetized for their better stability and solubility. Some of them were found to be more potent than acronycine on cell lines models and *in vivo*. This is particularly the case of a benzo[*b*]-acronycine series developed by Prof. François Tillequin and collaborators [84].This compound, named S23906-1, was selected as the lead of this series for its interesting cytotoxic potential in cellular models [85] and its *in vivo* antitumor activity using many anti-tumor models [86,87]. Indeed, the S23906-1 showed a similar or better antitumor activity than the drugs usually used in clinic (paclitaxel, topotecan, vinorelbine) against the different evaluated model: lung (A549 and NCI-H460), colon (HCT-116 and HT-29) and ovarian cancer (IGROV1 and NIH:OVCAR-3) xenografted in immunocompromised nude mice [87].

The initial evidences for DNA binding of first acronycine derivatives [88] were then completed by the identification of S23906-1 mechanism of action as a DNA alkylation agent through covalent bonding to the NH_2_ group of guanines orientated in the minor groove of the DNA helix [27]. Interestingly, alkylated single-stranded DNA portions are generated upon S23906-induced DNA alkylation [30]. Such DNA destabilization is dependent on the stereoisomeric position of the reactive groups on the skeleton of the *cis*-racemate S23906-1 [89].

At the cellular level, S23906-1 increases cyclin E (but not cyclins A, D1, D2 or D3 levels) level in HT-29 cells [85], and induces an irreversible S-phase blockade of the cell cycle and apoptosis in numerous cancer cell types [85,86,90]. In this case, the S23906-1 induced-cell death occurs though the generation of replication dependent double strand breaks [91]. By contrast, at low dose, S23906-1 induces a reversible G2/M phase arrest of the cell cycle and subsequent mitotic catastrophe in HT-29 and HeLa cells. Treatment of these cells with S23906-1 is associated with a rapid increase of cyclin B1 levels within 1 hour, followed by an increase in Cdk1 activity at 32 hours post-treatment suggesting that the cells enter mitosis after DNA damage [92].

In term of potential chemoresistance, S23906-1 not only reacts with DNA on the electrophilic NH_2_ group of guanines but also with the thiol group of cysteines present in small bionucleophiles such as glutathione (GSH) and *N*-acetyl-cysteine (NAC) in a manner that is associated with a reduction of its anti-proliferative activity [93].

Furthermore, in terms of DNA repair, alkylation by S23906-1 is associated with different DNA repair processes such as NER machinery implicating XPA, XPC and CSB (*Cockaine Syndrome* protein B) proteins [94], the global protein sensor ATM and Rad3-related kinase [95] and more recently with BRCA2 protein implicated in homologous recombination (HR) repair [96].

**Figure 3:**
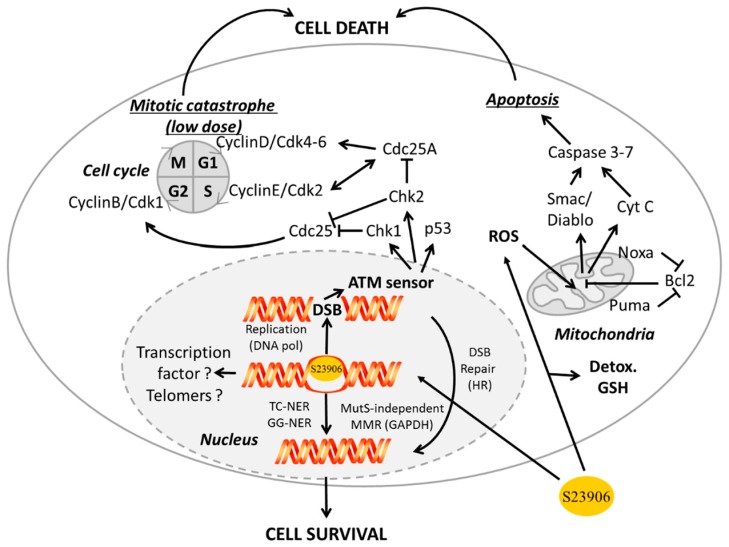
General scheme of S23906-1 cellular mechanism of action and repair. ROS, reactive oxygen species; CytC, cytochrome C; Detox, detoxification process; DSB, double strand break; DNApol, DNA polymerase; TC-NER, transcription-coupled nucleotide excision repair; GG-NER, global genome nucleotide excision repair; MMR, mismatch repair; HR, homologous repair. Solid and dash circles represent the cellular membrane and the nucleus, respectively. M, G1, S and G2 correspond to the different phases of the cell cycle. Open arrows and stop arrows correspond to activation and inhibition, respectively.

A propose global scheme is presented in Figure 3.

### 2.2. GAPDH

GAPDH was originally identified as a protein involved in glycolysis. Based on this general cellular function, GAPDH is supposed to be ubiquitously expressed [97,98] and is therefore commonly used as an housekeeping gene for internal control in protein samples loaded on western blots or for quantitative analysis of mRNA expression using PCRs. However, its function is much more complex and diverse than it was previously thought and GAPDH could no longer be considered as a good internal control for multiple cell normalization because of its over-expression in some models [99,100,101]. Excellent reviews are already published on the various GAPDH functions [102,103,104,105] with increasing knowledge on either neurodegenerative diseases [106,107,108] or cancer implication [109,110,111,112,113].

**Figure 4:**
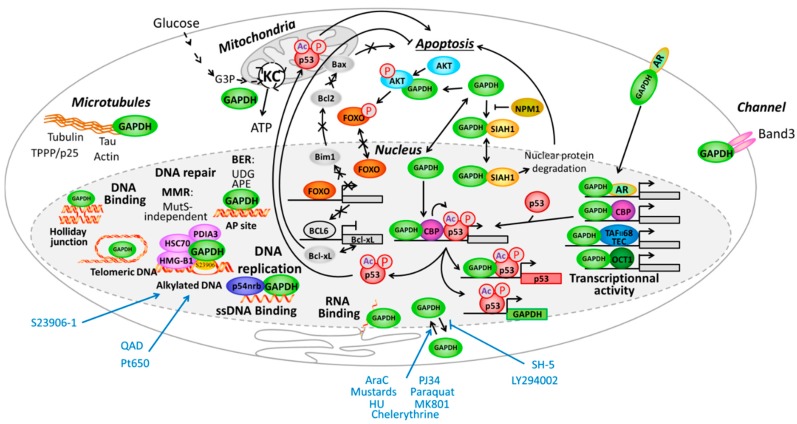
Illustration of some of the multiple functions of glyceraldehyde-3-phosphate dehydrogenase (GAPDH) presented in this review. KC, Krebs cycle; HU, hydroxyurea; Ac, acetylation; P, phosphorylation.

Within cell, GAPDH is implicated in numerous cellular processes and really deserves to be called a “moonlighting” protein, a term highlighting a class of proteins that present multiple cellular functions for an identical polypeptidic chain [114,115,116]. Those processes are well depicted in reviews. Here is an overview of the extranuclear processes involving GAPDH (see also Figure 4):

- At the cytoplasmic membrane level: GAPDH massively localizes in erythrocytes. It interacts with Band3 channel protein [102] or is implicated in endocytosis in CHO (Chinese hamster ovary) cells cells [117] and transferrin interaction in macrophages [118].
- In the cytoplasm: GAPDH was implicated in intracellular membrane trafficking, endoplasmic reticulum to Golgi transport and maintenance/regulation of protein polymerization. It interacts as a tetramer with tubulin to facilitate polymerization [119,120] and forms a complex with microtubule-associated protein 1B [121] or Rab2 [122]. Similarly GAPDH interacts with TPPP/p25 protein in Lewy bodies [123] and is found in neurofibrillary Tau proteins in brains from Alzheimer patients [124] and finally interacts with actin [125].
- In mitochondria: GAPDH is a glycolytic enzyme directly implicated in the 6th step of glycolysis to catalyze, in an NAD^+^-dependent manner, the conversion of glyceraldehyde-3-phosphate to d-glycerate-1,3-bisphosphate I to then be further converted in several steps to pyruvate that finally entered the Krebs cycle to produce energy.
- Associated with apoptosis through different ways:
  1. GAPDH interacts with phospho-AKT (P-AKT) to block its dephosphorylation, thus preventing Forkhead box class O protein (FOXO) nuclear translocation and further expression of the transcription inhibitor BCL6 that is usually responsible for inhibiting Bcl-xL expression. In that way the expression of anti-apoptotic protein Bcl-xL is reduce and apoptosis is enhanced upon control by GAPDH [126]. Since FOXO also activates Bim1, which controls apoptosis through Bcl2 and Bax, another way for GAPDH to potentially inhibit apoptosis is additionally presented in Figure 4.
  2. In parallel, interaction of GAPDH with P-AKT inhibits GAPDH nuclear translocation and subsequent acetylation/phosphorylation of p53 that further translocates in the mitochondria to initiate apoptosis [127].
  3. GAPDH forms a complex with the E3 ubiquitin ligase SIAH1. This GAPDH/SIAH1 complex translocated in the nucleus where it increases nuclear protein degradation associated with SIAH1 activity to further induce apoptosis [128,129]. This cascade could be either activated by paraquat [130], or inhibited upon S-nitrosylation of GAPDH by nucleophosmin (NPM1) [131]. Interestingly, both GAPDH and SIAH1 expression are controlled by p53 [132,133].
- Receptor mediated cell signaling, such as for the androgen-receptor that forms a complex with GAPDH to be then translocated to the nucleus [134]. This is also the case for interaction of GAPDH with the macrophage transferrin receptor, which forms a complex that is then translocated to the endosome compartment [135].

Since many reviews already presented those cytoplasmic functions of GAPDH, we will only focus on the nuclear functions of GAPDH in the following sections.

#### 2.2.1. GAPDH at the Interface of Nucleus and Cytoplasm Compartments

GAPDH translocates from cytoplasm to nucleus and vice-versa in association with different cellular processes [115,136,137].

First, GAPDH was implicated in tRNA binding [138] in the nucleus and to its export from nucleus to cytoplasm. GAPDH is also involved in mRNA stability of several genes among which are the endothelin-1 [139] the angiotensin II type 1 receptor (AT1R) [140], the colony-stimulating factor 1 (CSF-1) [141], the CCN2/CTGF (connective tissue growth factor) [142] and the cyclooxygenase 2 genes [143]. For instance, GAPDH binds to an 84 nucleotides region called CAESAR, located in the 3′-untranslated region of CCN2 mRNA, and stabilizes it, but only in hypoxic conditions and in the absence of NAD^+^ [142]. Similarly, binding of GAPDH to the 144 bases region, named ARE for AU-rich element, in CSF-1 mRNA stabilizes it [141]. By contrast, GAPDH binds to another ARE located in the 3′-UTR region of COX2 mRNA through its NAD^+^-interaction domain and favors its degradation [143]. GAPDH also binds to the ARE of TNF-α mRNA in a two-step manner with a first GAPDH tetramer binding and second tetramer interaction that stabilizes the complex [144].

Second, GAPDH itself translocates in the nucleus upon different cell stimuli such as the treatment of cells with aracytin (AraC). As a consequence, it interacts with nuclear macromolecules as evidenced from a reduced mobility of GAPDH-GFP fusion protein within the nucleus [145,146] in a manner that is associated with chemosensitivity to AraC [147]. As part of an activation loop, AraC itself induces an increase in GAPDH expression through p53 [132]. The nature of the isoforms of GAPDH transferred to the nucleus was further identified as six different nuclear GAPDH isoforms with isoforms 1 to 3 being the most frequent in the nucleus but the minor isoforms 4 to 6 being the first to be increased after only four hours of AraC treatment [148]. GAPDH translocation is also achieved under treatment with hydroxyurea and chelerythrine [149,150] as well as with the herbicide paraquat [130]. Treatment with sulfur mustard also induces GAPDH nuclear translocation, more precisely of the basic GAPDH isoform that presents an apparent isoelectic point pI of 8.5 [151]. Functionally, GAPDH accumulation in the nucleus is also associated with apoptosis pathway upon complex with SIAH1 protein upon activation by glutamate or kinases such as the signal-regulating kinase 1 (ASK1) [152]. GAPDH/SIAH1 complex then enters the nucleus [153]. Nuclear translocation is also associated with a CRM1-mediated nuclear export signal [154].

Third, GAPDH translocation to the nucleus could be inhibited by deprenyl, a neuroprotectant used in Parkinson’s disease [155], the PARP inhibitor PJ34 [156] or both the *N*-Methyl-d-aspartate receptor antagonist MK801, the neuronal nitric oxide synthase inhibitor 7-nitroindazole and monoamine oxidase-B inhibitor (R)-(-)-deprenyl hydrochloride [157]. GAPDH nuclear translocation is also inhibited by cellular proteins such as glutaredoxin 1 [158], CIB1 [159], the mitochondrial uncoupling protein 2 (UCP2) [160], SIRT1 [153], GOSPEL [161] or the protein kinase B-β (AKT2) [162], in association with inhibition of its apoptotic activity or neuroprotective activity. As a consequence, nuclear export of GAPDH is inhibited by the phosphoinositide 3-kinase (PI3K) LY 294002 and AKT inhibitor SH-5 [163] (Figure 4).

#### 2.2.2. GAPDH Modulates Transcription Factor Activity

First insight in GAPDH function associated with transcription came for its identification as a single-strand DNA binding protein (SSBP) and the observation that it activates RNA polymerase II in *Xenopus leavis* ovocytes [164]. GAPDH was then found to interact with the oncogenic fusion proteins hTAF_(II)_68-TEC generated from t(9;17) translocation associated with extraskeletal myxoid chondrosarcomas [165].

By interacting with p300/CBP (CREB binding protein), GAPDH is acetylated on Lys162 in a nitric oxide (NO) dependent way. In return, GAPDH stimulates self- acetylation of PBC [166], which then activates p53 and induce cell death [167].

Nuclear GAPDH also directly interacts with OCT-1 transcription factor within the OCA-S complex. This multicomponent protein complex regulates the expression of several genes among which the histone H2B expression associated in DNA replication [168,169].

Similarly, GAPDH complexes with p53 transcription factor to then transcriptionally activates different genes among which the p53 itself [170] (Figure 4).

#### 2.2.3. GAPDH and DNA Binding

GAPDH was evidenced as RNA binding protein, affecting the stability and/or conformation of mRNA and tRNA (see above) but it could also bind to DNA: single- or double-stranded DNA, alkylated DNA as well as triplex or quadruplexes DNAs.

Some post-translational modifications of GAPDH favor its interaction with nucleic acids. For instance, the oxidation of various thiol groups favor changes in the stoichiometry of GAPDH from monomeric, to dimeric or tetrameric form [171]. Glutathionylation and nitrosylation of GAPDH, involved in protein nuclear import, are also potential mediators of GAPDH/DNA interaction. Indeed, gel shift experiments showed that each of these isoforms interacted with a DNA fragment of 248 pb [172]. Other modifications may come from genotoxic or alkylating drugs, such as the diepoxybutane, which react with the Cys246 residue of GAPDH and thus promotes GAPDH interaction with DNA [173].

Besides single- and double-strand DNA binding, GAPDH interacts with telomeric DNA [174,175,176]. This was well illustrated in A549 lung cancer cells treated with doxorubicin and gemcitabine [174]. *In vitro*, GAPDH binds as a dimer or a tetramer on single stranded telomeric sequences through its Asp34 and Cys152 residues [175]. Mutation of either of these sites inhibits GAPDH/telomeric DNA interaction, without preventing nuclear translocation, thus resulting in a shortening of the cellular telomeric sequences followed by cell cycle arrest [175]. As previously observed with RNA, the DNA binding requires the NAD^+^-binding site of GAPDH. Overexpression of GAPDH also prevents telomere degradation after exposure of cells to ceramide [174]. The recognition of telomeric sequences by GAPDH is not specific to cancer but is also found in the *Trypanosoma cruzi* parasite [177]. In cancer, the interaction of GAPDH to telomers leads to chemoresistance and apoptosis [175] or senescence [178]. In yeast, interaction of the GAPDH isoform Tdh3 with the NAD^+^-dependent histone deacetylase Sir2 is even implicated in transcriptional silencing of telomeres (and ribosomal DNA) [179].

Besides the recognition of native DNA and RNA, GAPDH strongly recognizes thiopurines and is implicated in its DNA repair (see Section 2.2.5.). GAPDH also binds as a monomer to some DNA adducts formed using the saframycin A derivative QAD and the ET-743 derivative Pt650. Different cellular consequences were identified depending on the organisms in which it was evaluated. For instance, saframycin A or QAD treatment of yeast increased the expression of TDH1, -2 and -3 genes, three yeast isoforms of GAPDH [180]. By contrast, nuclear translocation of GAPDH was observed after a 48 h treatment with QAD in the HeLa-S3 human cells, whereas human lung carcinoma cells A549 treated with an siRNA directed against GAPDH are more resistant to QAD, suggesting that GAPDH could be involved in the cytotoxic activity of QAD in this model [181].

More recently, direct interaction of GAPDH was evidenced with apyrimidic/apuric (AP) sites in double stranded DNA. The AP site recognition was followed by a stable crosslink via β-elimination to form an adduct through thiol oxidation of GAPDH without AP lyase activity to further remove AP site and generate a strand break [182].

#### 2.2.4. GAPDH and DNA Replication

Some evidence of GAPDH interference with DNA replication came from the preferential interaction of GAPDH binding to single-stranded DNA (ssDNA) with a better affinity than that of DNA polymerase α, thus resulting in an inhibition of the DNA synthesis [183].

Topoisomerase I is implicated in DNA replication process. Upon H_2_O_2_ treatment of HEK293T cells, GAPDH is oxidized and consequently interacts with the p54nrb/PSF (polypyrimidine tract-binding Splicing Factor associated protein) complex. This protein p54nrb is involved in multiple nuclear functions and its interaction with PSF noteworthy increases the topoisomerase I induced DNA relaxation activity [184].

#### 2.2.5. GAPDH Implication in DNA Repair

GAPDH was first implicated in Base Excision Repair (BER). This pathway is particularly involved in the repair of oxidized bases and some damaged bases generated from alkylating agents such as methylating drugs [185]. BER can process the damaged bases through two repair mechanisms: “short-patch”- and “long-patch”-BER. They both start with the recruitment of a DNA glycosylase presenting single-stranded or double-stranded DNA binding properties, as for the uracyl DNA glycosylase (UDG), to generate an abasic site (AP). The APymidic/APuric Endonuclease 1 (APE1) then cleaves the generated phosphodiester bridge to form a single strand break (SSB) in the DNA double helix. This SSB is recognized by the polyADP-ribose polymerase (PARP) that adds ADP-ribose groups to mark damaged DNA and makes it accessible to repair proteins such as XRCC1, but also prevents the binding of DNA polymerase α [186].

GAPDH monomer possesses an intrinsic UDG activity [187,188] that was however less active than that of the *Escherichia coli* UDG enzyme [102]. GST pull-down and co-immunoprecipitation experiments showed moreover that GAPDH physically interacts with APE1. In presence of H_2_O_2_, GAPDH reduces the oxidized form of APE1 through its residue Cys152 and subsequently decreases its endonuclease activity [189]. Similarly stress-induced DNA damage results in an increased level of polyphosphate diadenine (Ap4A) that is then recognized by PARP [190], but also by GAPDH/UDG in HeLa cells [191]. As indicated above, direct covalent interaction of GAPDH with AP site was very recently evidenced that could participate in DNA repair process maybe as a damage sensor or damage protection mechanism that needs to be further investigate [182].

GAPDH was also implicated in mismatch repair (MMR) pathway, and more precisely in the MutSα-independent MMR process (Figure 4). Briefly, MMR recognizes a mismatched bases caused for instance by replication errors, or abnormal bases such as thioguanosine [192]. Treatment with mercaptopurine of lymphoblastic leukemia cell lines lacking or overexpressing some proteins involved in MMR repair pathway showed that the sensitivity of cells to treatment to mercaptopurine was independent from expression of MutSα proteins. Krynetski *et al.* [193] highlighted a new DNA repair pathway involving a protein complex comprising HMG-B1, HMG-B2, HSC-70, ERp60 and GAPDH and that was independent of the classical MMR and MutSα. Within this five proteins complex, HMG-B1 seems to interact directly with DNA at thioguanosine residue and the authors suggests that GAPDH binds more likely at the periphery of the complex since it could be removed easily from this complex. The same results were obtained upon treatment with 5-fluorouracil (5-FU) for which an increased expression of the GAPDH was also observed [194]. A similar complex was implicated in the cellular consequences of AraC treatment [195].

### 2.3 GAPDH Binds to S23906-1/DNA Adducts and Affects Its Cytotoxic Activity

**Figure 5:**
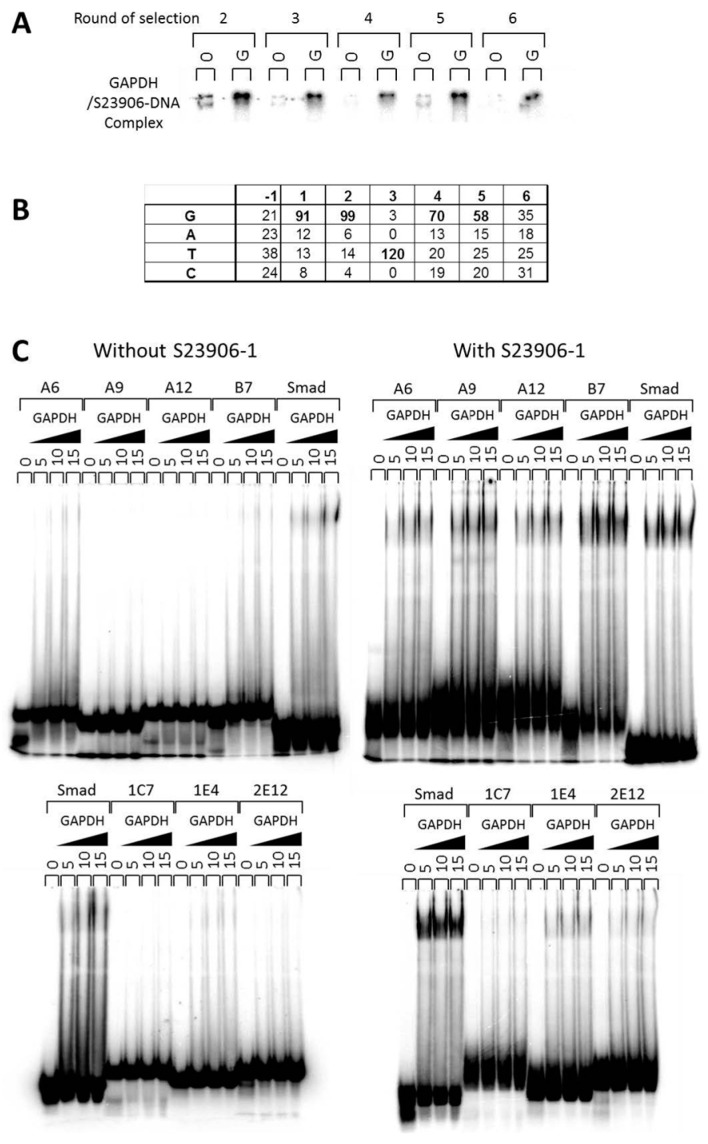
Binding of glyceraldehyde-3-phosphate dehydrogenase (GAPDH) to S23906-1 DNA adducts. (**A**) Increased selectivity for adduct recognition in the course of binding site selection is visualized upon migration of the radiolabeled mixture of DNA with GAPDH (5 μg) on a native 6% polyacrylamide gel. “0” and “G” refer to the absence or presence of GAPDH proteins; (**B**) Determination of the preferential binding site upon alignment of the amplified sequences; (**C**) EMSAs evidence a differential complexation efficiency of GAPDH on S23906-1 alkylated (labeled “with S23906-1”) or unalkylated (labeled “without S23906-1”) using various DNA sequences from [176] and comparison with the reference DNA Smad-SBE (Smad).

In the particular case of the DNA destabilizing adduct S23906-1, the nuclear GAPDH enzyme was found to directly bind to the generated DNA adduct [176]. Indeed, in order to search for proteins that specifically recognize the S23906-1/DNA adduct using chromatography column, GAPDH and HMG-B1 proteins were identified by MALDI-TOF spectrometry of proteins separated by SDS-PAGE. Both purified proteins were tested using EMSA for direct binding to this adduct but only GAPDH presented a direct interaction. The specific sequence for adduct recognition was investigated using a SELEX (Systematic Evolution of Ligands by Exponential amplification) based approach of six round of selection (Figure 5A) to be the consensus sequence GGT(G/t)(G/t) as evidenced from sequencing of individually subcloned oligonucleotides (Figure 5B). The abilities of various representative sequences were then validated using EMSA for both native and alkylated oligonucleotides ([176] and Figure 5C).

**Figure 6:**
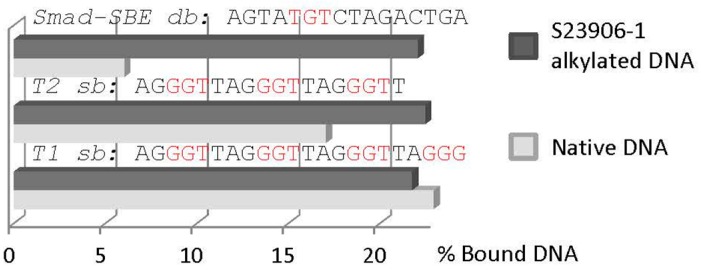
Histogram of the quantification of GAPDH bound to S23906-1/DNA adducts or native DNA. Two single stranded telomeric sequences were used (T1 and T2) in comparison with the double-stranded Smad-SBE oligonucleotide for binding to the same amount of GAPDH.

In parallel, we searched for potential inhibition of transcription factor/DNA binding inhibition using HeLa nuclear extracts and Transignal Protein/DNA Arrays from Panomics but also evaluated the impact of GAPDH binding to this pool of alkylated transcription factor binding sequences as a mixture of S23906-1 alkylated DNA sequences. Representative results were presented in Figure 5 and Figure S1 of reference [176]. Such analyses evidenced that GAPDH binds more strongly to alkylated Smad-SBE sequence rather than non-alkylated one (Figure 5C and unpublished data). Such enhancement was not obtained using telomere sequences of three (T2) or four (T1) guanine stretches for which GAPDH binding is similar between alkylated and non-alkylated DNAs (Figure 6).

**Figure 7:**
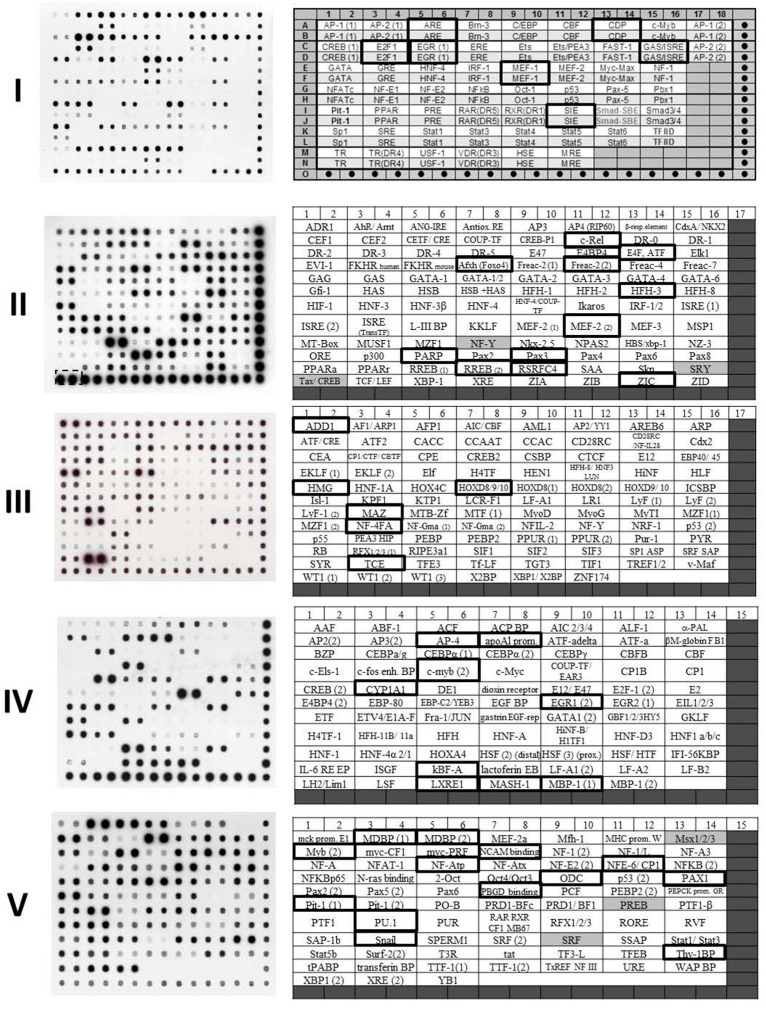
Transignal protein/DNA arrays using GAPDH and unalkylated DNA oligonucleotides. The experiments were performed as presented in [176]. In Transignal membrane I (labeled “I”), dot-containing boxes correspond to hybridization controls and the target sequences for each transcription factor are spotted four times: the two upper spot being 10 fold more concentrated (white boxes) than the two lower ones (light grey boxes). In Transignal membrane II to V (labeled “II” to “V”), the hybridization controls are localized in dark grey boxes and the target sequences for each transcription factor are spotted in duplicate at equal concentrations. Positions identified using black rectangles correspond to those for which a stronger GAPDH/DNA complex could be identified. The corresponding DNA sequences are presented in Table 1.

Particularly, this work identified the Smad/SBE sequence as a target for GAPDH specific binding to S23906-1 alkylated DNA. By taking opportunity to further investigate here the impact of GAPDH binding to unalkylated DNA using Transignal protein/DNA arrays I to V that were done in parallel as controls, it was possible to appreciate the potential specific binding to native double strand DNA of various sequences. Incubation of 1 μg of GAPDH with the mixture of transcription factor DNA binding oligonucleotides (corresponding to 393 different sequences) was performed as described in [176]. The results are presented in Figure 7 and Table 1. Highlighted in Figure 7 using bold rectangles are the strongest complexes formed between GAPDH and the corresponding DNA sequence and highlighted in grey in Table 1 are the corresponding oligonucleotide sequences that contains the consensus sequence GGT(G/t)(G/t) previously identified using S23906-1 alkylated DNA [176]. This consensus is not found in all sequences. The closest sequences found in those oligonucleotides are underlined in Table 1 and different oligonucleotides that do not share any homologies are even bound by GAPDH (see for instance GAS/ISRE, c-Rel, HMG, MDBP (1) or NCAM-BP sequences), thus reinforcing the idea that GAPDH has a poorest DNA binding selectivity than most transcription factors.

The transcription factor DNA binding sequences are here used as a mixture of a variety of DNA sequences. However, it would be interesting to further evaluate the incidence of GAPDH interaction to some highly recognized sequences in order to precise if GAPDH covering sequences match the identified transcription factor binding site and if GAPDH could compete for this transcription factor activity at the molecular and the cellular level. This would be particularly interesting for MEF-1 protein which recognizes the 5′-CAGGTG consensus sequence (in bold and italics) [196] that is partially covered by the identified sequence for GAPDH binding (underlined) in the oligonucleotide 5′-TCAGGCAG***CAGGTG***TTGGGGGGAT.

Such potential effect on transcription factor activity might then be another function of the moonlight protein GAPDH.

**Table 1:** Sequences of the bound oligonucleotides. The name and sequences of oligonucleotides correspond to that that formed a GAPDH/DNA complex formed a revealed using Transignal membranes presented in Figure 7. In grey: sequences that contains the 5′-GGT(G/T)(G/T) site. Underlined: potential GAPDH binding sites from comparison with sequences obtained from SELEX. “+” and “++” refers to strong and very strong binding of GAPDH to DNA at the indicated sequence.

TranSignal Membrane	Transcription Factor	GAPDH Binding	Sequence for TranSignal Oligonucleotides Containing Transcription Factor Consensus Sites
I	ARE	+	GTCTGGTACAGGGTGTTCTTTTT
	CDP	+	TCAGAAATTGGCTAATAATCATTGGG
	E2F1	++	ATTTAAGTTTCGCGCCCTTTCTCAA
	EGR(1)	+	GGATCCAGCGGGGGCGAGCGGGGGCCA
	GAS/ISRE	+	CGAAGTACTTTCAGTTTCATATTACTCTACAA
	MEF-1	++	TCAGGCAGCAGGTGTTGGGGGGAT
	SIE	+	GTGCATTTCCCGTAAATCTTGTCTACA
II	c-Rel	+	GGGGATTTCCGGGGATTTCCGGGGATTTCC
	E4F/ATF	+	GGCTGACGTCACTGGGCTGACGTCACTG
	AFXH (FOXO4)	+	GTTGTTTATGGTTGTTTATGGTTGTTTATG
	Freac2-2	+	TTGTTTTGTTGTTTTGTTGTTTTG
	HFH3	+	GGGTGTTTGTTTAGGGTGTTTGTTTA
	MEF-2 (2)	++	GCTATTTTTAACGAGGGCTATTTTTAACGAGG
	PARP	++	ATGGGAGGGGCAATGGGAGGGGCA
	PAX3	+	GATCCTGAGTCTAATTGGATCCTGAGTCTAATTG
	REBB2	++	TGGAAATGGCGGGGGATGGTGGGGGACCGGATC
	RSRFC4	+	GGTCTATTTATAGCTTGGTCTATTTATAGCTT
	ZIC	+	CATAGTTTCTAAAAGAGGAGGAGGTAGTTCTAG
III	ADD-1	+	TCCTAGTGTGAGCGGCCCT
	HMG	+	CGATCTGGAACTCCGGGAATTTCCCTGGCCC
	HOXD-8/9/10	+	GCGGCAGTTTTATTGTTTTATTCGC
	MAZ	+	GGGTTGGGGAAGTATTAGGAGGGGAGGGTT
	NF-4FA	+	CTCCTTTCTTTGAAGCTCCTTTCTTTGAAG
	TCE	++	GCAGAGGGCGTGGGGGAAAAGAA
IV	AP-4	+	TCAGCGCGGGTCAGCGCGGGATTC
	ApoA1Prom	++	CCCTGCAAGAGCTGGCTGCTTAGAGACTGCGAGAAGGAG
	c-Myb (2)	+	GGACCAGGGGGTCTAGGAG
	CYP1A1	++	GTAAGGGGGCAGAGGTCGGG
	EGR1 (2)	++	CCTCCCCCCGCCTTGCCCGGGGTTGTGG
	kBF-alpha	+	GGCGTTTTCGTTTTTACCCGGC
	LXRE-1	+	GCTGAGGTTACTGCTGGTCATTCAAGCT
	MASH-1	+	GGCTCAGGCAGCAGGTGTTGGG
	MBP-1 (1)	+	GTGGGAAATTCCGTGGGAAATTCC
V	MDBP (1)	++	CTATTGGCGTTACTATGGGAACATA
	MDBP (2)	+	GGCCATTACCTGGTGATATTACCTGGTGATGC
	Myb (2)	+	GCCCAGTTGTTAGCCCAGTTGTTA
	NCAM-BP	++	GCTCTGCATTTTCTTTTGGCC
	NF-Atp	+	TTGCATTTTCCATGGTTGCATTTTCCATGG
	NF-E6/CP1	+	ACTGAGTCATGAGTCATGGTTGGCCC
	ODC	+	TGCGTCTCCATGACGGTCTCCATGACGAC
	PAX-1	+	CACCGTTCCGCTCTAGATATCTC
	PBGD BP	+	TCAGTGTCCTGGAGTGTCCTGGTTACT
	Pit-1 (1)	+	CTAAATTATCCATTTATCCATTAGCAC
	PU.1	+	AGAAAAGGAGAAGTAGGAGGC
	Snail	+	TGTGAACAGGTGCTTGTGAACAGGTGCT
	Thy-1 BP	+	GATCAGGGGTGGCAGGGGTGGAAT

As part of the present function of GAPDH to recognize S23906-1/DNA adducts, the cellular consequences were evaluated in cell lines presenting different p53 status. Indeed, in the p53 wild-type cell line A549, which survival is affected by a decrease of GAPDH using siRNA [147], increasing GAPDH expression is associated with an increased S23906-induced apoptosis and a decreased cell survival [176]. By contrast, in the p53-mutated cell line HT-29, which cell survival is much lesser affected by GAPDH expression, silencing of GAPDH increases S23906-induced cytotoxicity, suggesting a protective effect of GAPDH on S23906-induced cell death. If GAPDH binding to chromatin is increased upon DNA alkylation by S23906-1 in both A549 and HT-29 cells, a strong increase of HMG-B1 binding to chromatin (in correlation with a decrease in the HMG-B1 proportion in the nuclear soluble fraction) is only seen in HT-29 treated cells whereas HSC70 binding is increased in both cell lines. As presented in the previous section, GAPDH, HMG-B1, HSC70, and PDIA3/ERp57 proteins are implicated in a complex as a MutS-independent MMR repair machinery [193,194] and could therefore be implicated in S23906-1/DNA adducts repair in the p53-mutated cell line (Figure 4). The pro-apoptotic effect of GAPDH in p53-WT cell line A549 found using S23906-1 is similar to that observed by others with treatment with AraC upon which HMG-B1 and PDIA3 induces phosphorylation of p53 and γH2AX, respectively, in response to AraC-induced DNA damages [195]. HMG-B1 itself was also implicated in AraC, adriamycin and vincristine sensitivity in the Jurkat (T-ALL) and K562 (CML) leukemia models [197].

## 3. Conclusions

The present review aims at presenting the importance of DNA adduct recognition by several proteins from the “attempted” DNA recognition machinery: DNA repair, transcription, and replication processes but also by unexpected proteins such as the moonlight protein GAPDH. Besides its multiple well described cytoplasmic and nuclear functions, GAPDH also recognizes several DNA adducts, among which the S23906-1/DNA complexes presented here as an interesting example. The adduct recognition occurs whatever the DNA form is: single-stranded, double-stranded or telomeric DNAs. Such binding could have a multitude of cellular consequences on DNA replication, transcription, repair, and stability that would be interesting to further evaluate. Since GAPDH proteins are subjected to large variety of post-transcriptional modifications (phosphorylation, acetylation, *S*-nitrosylation, glucorinylation, oxidation, ADP-ribosylation, carbonylation, acrylamide adducts, O-linked *N*-acetylglucosamine acylation, *S*-glutathionylation, *S*-nitrosoglutathionylation, *etc.*) [113] and could act as monomer, dimer or tetramer, the multiplicity of the GAPDH protein isoforms need to be further clarified in relation to the diversity of its cellular function.

Finally, in terms of drug development, the precise analysis of the mechanism of action of a new drug candidate would contribute to pave the way for personalized medicine, as is suggested here for S23906-1, for which the implication of GAPDH would lead to opposite cellular effect depending on p53 status [176].
